# In situ stoichiometry amounts of p62 and poly-ubiquitin exceed the increase of alpha-synuclein during degeneration of catecholamine cells induced by autophagy inhibition in vitro

**DOI:** 10.1007/s00702-024-02795-x

**Published:** 2024-06-18

**Authors:** Paola Lenzi, Gloria Lazzeri, Michela Ferrucci, Carla Letizia Busceti, Stefano Puglisi-Allegra, Francesco Fornai

**Affiliations:** 1 Department of Translational Research and New Technologies in Medicine and Surgery, University of Pisa, Pisa, Italy; 2 IRCCS, Istituto di Ricovero e Cura a Carattere Scientifico, Neuromed, Pozzilli, IS Italy

**Keywords:** Sequestosome, Autophagosome, Autophagy, Proteasome, Lewy bodies, Poly-ubiquitin

## Abstract

Neurodegenerative disorders are typically featured by the occurrence of neuronal inclusions. In the case of Parkinson’s disease (PD) these correspond to Lewy bodies (LBs), which are routinely defined as proteinaceous inclusions composed of alpha-synuclein (alpha-syn). In turn, alpha-syn is considered to be the key protein in producing PD and fostering its progression. Recent studies challenged such a concept and emphasized the occurrence of other proteins such as p62 and poly-ubiquitin (Poly-ub) in the composition of LBs, which are also composed of large amounts of tubulo-vesicular structures. All these components, which accumulate within the cytosol of affected neurons in PD, may be the consequence of a dysfunction of major clearing pathways. In fact, autophagy-related systems are constantly impaired in inherited PD and genetic models of PD. The present study was designed to validate whether a pharmacological inhibition of autophagy within catecholamine cells produces cell damage and accumulation of specific proteins and tubulo-vesicular structures. The stoichiometry counts of single proteins, which accumulate within catecholamine neurons was carried out along with the area of tubulo-vesicular structures. In these experimental conditions p62 and Poly-ub accumulation exceeded at large the amounts of alpha-syn. In those areas where Poly-ub and p62 were highly expressed, tubulo-vesicular structures were highly represented compared with surrounding cytosol. The present study confirms new vistas about LBs composition and lends substance to the scenario that autophagy inhibition rather than a single protein dysfunction as key determinant of PD.

## Introduction

In neurodegenerative disorders such as Parkinson’s disease (PD), neuronal loss is often accompanied by the accumulation of protein-containing and non-protein-containing structures. This typically occurs according to a concentration gradient within discrete cell domains of spared neurons (Forno 1996; Iwatsubo et al. 1996; Shahmoradian et al. 2019; Ferrucci et al. 2024; Lenzi et al. 2024), which sometime grow up significantly to produce frank cytosolic inclusions. In PD, these neuronal inclusions are eosinophilic and they are named Lewy bodies (LBs), being considered routinely as a hallmark of disease (Lewy 1912; Tretiakoff 1919; Forno 1996; Iwatsubo et al. 1996; Spillantini et al. 1997; Shahmoradian et al. 2019; Lashuel 2020; Ferrucci et al. 2024; Lenzi et al. 2024). When analyzing LBs or smaller abnormal cytosolic eosinophilic domains, it is important to decipher both their structure and formation dynamics. In fact, these are likely to be seminal points to comprehend the neurobiology of PD, to identify disease markers and to develop specific disease-modifying therapies in PD (Forno 1996; Fornai et al. 2005; Jellinger 2014; Lashuel and Novello 2021; Lenzi et al. 2024; Wang et al. 2024). Although routine definition of LBs refers to alpha-syn aggregates (Spillantini et al. 1997, 1998; Lashuel 2020; Lashuel and Novello 2021; Estaun-Panzano et al. 2023; Wang et al. 2024), classic and recent evidence emphasizes a conspicuous presence of proteins other than alpha-syn along with noticeable amounts of non-protein structures when analyzed ex vivo from PD and Dementia with Lewy Bodies (DLB) patients or from cell cultures in vitro (Forno 1996; Iwasubo et al. 1996; Shahmoradian et al. 2019; Lashuel 2020; Estaun-Panzano et al. 2023; Lenzi et al 2024). So far, the quantitative contribution of each specific component to cast LB structure remains to be established including the authentic amount of alpha-syn. In fact, no study established in situ the specific amount of alpha-syn compared with other proteins and non-protein structures (Lenzi et al. 2024). This is due to a lack of quantitative studies measuring in situ stoichiometry amount of alpha-syn and other specific proteins as well as the number of specific vesicular organelles. Thus, the pivotal role of alpha-syn albeit routinely assumed, remains to be validated by the gold-standard procedure applying in situ stoichiometry to count immunogold particles (Bergensen et al. 2008). Indeed, some experimental and pathological data are challenging a pivotal role of alpha-syn. In fact, experimental eosinophilic LB-like inclusions induced by proteasome inhibition may develop as p62 and poly-ubiquitin (Poly-ub) eosinophilic bodies in the absence of alpha-syn (as shown in alpha-syn knocked out mice, Paine et al. 2013). In line with this, some evidence emphasizes a primary role of other proteins such as p62 and Poly-ub (Forno 1996; Iwasubo et al. 1996; Komatzu et al. 2007; Geisler et al. 2010; Sato et al. 2018, 2021). Recently, these proteins were suggested to seed LBs in experimental parkinsonism and some genetic PD (Komatzu et al. 2007; Hattori and Mizuno 2017; Sato et al. 2018; 2020; Gao et al. 2022; Noda et al. 2022; Oh et al. 2022; Ferrucci et al. 2024; Lenzi et al. 2024). In keeping with the hypothesis that other proteins play a significant role in composing and seeding LBs, it appears that, in vitro injection of pure alpha-syn aggregates, although being a *bona fide* approach, is unlikely to produce LBs mimicking the natural cytopathology of PD. Again, non-protein structures are recently claimed to be able to seed LBs and generate LBs maturation through an auto-catalytic mechanism (Cholak et al. 2019; Kuznetsov and Kuznetsov 2022; Kuznetsov 2024). Thus, when considering various components (proteins, organelles and less defined membranous structures), which compose a LB, it is difficult to identify a single protein or a single organelle as the sole guilty and precise disease-determinant, which triggers either LB formation or PD progression (Shahmoradian et al. 2019; Lenzi et al. 2024). Accordingly, recent studies trying to decipher the natural cytopathology of PD emphasize the failure of cell clearing pathways rather than specific protein or lipid structures as disease determinants. Within this novel scenario a metabolic machinery named autophagy seems to be strongly involved (Anglade et al. 1997; Komatzu et al. 2007; Ferrucci et al. 2008; Pasquali et al. 2009; Isidoro et al. 2009; Lenzi et al. 2012; Liu et al. 2019; Vidyadhara et al. 2019; Sato et al. 2020; Senkevich and Gau-Or 2020; Oh et al. 2022; Smith et al. 2023; Yang et al. 2023; Zhu et al. 2023; Dong et al. 2024; Gu et al. 2024; Hull et al. 2024). In fact, when conditional knock out mice for the autophagy gene ATG7 are generated, neuronal death occurs within *substantia nigra pars compacta* (SNpc), where spared neurons develop LB-like neuronal inclusions (Sato et al. 2018). In these mice LB-like neuronal inclusions are seeded at first by p62 and Poly-ub, while alpha-syn adds later on (Sato et al. 2018). This replicates data obtained by Komatzu et al. (2006, 2007), who generated LB-like inclusions through genetic autophagy inhibition by silencing ATG7. Komatzu’s findings highlight both the relevance of autophagy inhibition and the early presence of p62 in the process of LB formation. The same results were produced by Ahmed et al. (2012) in a conditional mouse model, where ATG7 was selectively knocked out within mesencephalic dopamine (DA) neurons. In these mice, at short time interval, a moderate increase in low molecular weight alpha-syn was documented, while LBs were characterized by the presence of p62/Poly-ub without a concomitant alpha-syn immunostaining (Ahmed et al. 2012). Similarly, an increased amount of a transcription factor regulating gene expression named miR-214–3p, which suppresses the expression of autophagy genes produces experimental PD due to autophagy failure, which is manifest along with a massive increase in p62 (Dong et al. 2024). When looking at this scenario the role of alpha-syn in PD patients may be slightly toned down. This is in line with recent evidence by Oh et al. (2022), which indicates that degradation of p62 through S-nytrosilation is seminal to induce autophagy inhibition, and subsequent alpha-syn positive inclusions. In keeping with such a mechanistic hypothesis, we designed the present short study, where we administered various doses of the well-known autophagy inhibitor 3-methyladenine (3-MA). The study was designed to assess, whether a pharmacological autophagy inhibitor was analogous to a genetic autophagy inhibition in producing catecholamine cell damage accompanied by accumulation of alpha-syn, p62 and Poly-ub within catecholamine cells. In these experimental conditions we detected and compared for the first time the authentic amount of alpha-syn, p62, and Poly-ub in situ even considering non-protein tubulo-vesicular components. Thus, in simplified in vitro conditions we reproduced the autophagy inhibition within catecholamine cells to evaluate the amount of cell damage and to quantify through in situ stoichiometry specific proteins and non-protein structures, which characterize LB. These measurements were carried out at first through a rough evaluation of specific immunofluorescence and they were substantiated by authentic stoichiometry providing the number of molecules for each protein and the area of membranous structures analyzed in situ by transmission electron microscopy (TEM). This approach was pursued to get a deeper insight into arbitrary assumptions based on qualitative staining appearing in immunohistochemistry. Moreover, since some papers report that under specific experimental conditions 3-MA may also act as an autophagy activator (Wu et al. 2010; Klionsky et al. 2021), we measured the autophagy flux to assess whether 3-MA, at the dose and time interval used in these experiments is effective in inhibiting autophagy.

## Materials and methods

### Cell cultures

Pheochromocytoma PC12 cell cultures, purchased from IRCCS San Martino Institute (Genova, Italy), were kept in a wet atmosphere with 5% CO_2_ at 37 °C and they were grown in RPMI 1640 medium (Sigma-Aldrich, St. Louis, MO, USA), supplemented with horse serum (HS, Sigma-Aldrich), fetal bovine serum (FBS, Sigma-Aldrich), and antibiotics (streptomycin and penicillin). Experiments were carried out when PC12 cells were in the log-phase of growth, corresponding to 70% confluence (Qiao et al. 2001; Song et al. 1998). Before experimental treatments, cells were seeded according to the different experimental procedures and incubated for 24 h at 37 °C in 5% CO_2_. In detail, for light microscopy staining procedures, 1 × 10^4^ or 5 × 10^4^ PC12 cells were seeded in 24-well plates in a final volume of 1 mL/well. For TEM, 1 × 10^6^ cells were seeded in culture dishes in a final volume of 5 mL.

### Cell treatments

When cells reached the log-phase of growth, they were treated with the autophagy inhibitor 3-methyladenine (3-MA), which was dissolved in the culture medium at four concentrations (0.1 mM; 1 mM; 10 mM; 50 mM) to be used for histochemistry (H&E and TB) and histofluorescence (FluoroJade-B, FJ-B). Based on the effects induced by specific doses of 3-MA on cell integrity, a gold-standard dose of 3-MA of 10 mM for 72 h was selected to analyze the cells at immunohistochemistry and immunoelectron microscopy.

In experiments aimed at assessing the inhibition of autophagy, the autophagy inhibitor bafilomycin A1 (100 nM, Sigma-Aldrich) was added to the culture medium 3 h before the end of the treatments with 3-MA.

### Histochemistry and immunohistochemistry

#### Trypan blue (TB)

To assess the percentage of dying cells, following various doses of 3MA, TB staining was carried out. Twenty-four hour before treatment, 1 × 10^4^ PC12 cells were placed within 24-well plates in 1 mL culture medium without any fixing procedure. After treatment with various doses of 3-MA, cells were collected and centrifuged at 800×*g* for 5 min, cell pellets were suspended in 0.5 mL of culture medium, and 25 µL of cell suspension were incubated, for 5 min in a solution of 1% TB (62.5 µL) and PBS (37.5 µL). Ten µL aliquot of this solution were analyzed using a Bürker chamber under Olympus CKX 41 inverted microscope (Olympus Corporation, Tokyo, Japan). Viable and non-viable cells were counted, and cell death was expressed as the mean percentage ± SEM of TB-positive cells out of total cells. For each treatment group data are expressed as the mean of three chambers counts, which were replicated for three independent experiments and counted by two blind investigators.

#### Further histochemical techniques (H&E and FJ-B) and immunohistochemistry

All these procedures are described apart since, differing from TB, they were carried out on paraformaldehyde fixed cells. In detail, 5 × 10^4^ PC12 cells were seeded on poly-lysine coverslips and placed in 24-well plates (final volume: 1 mL/well). These cells were fixed with 4% paraformaldehyde in PBS for 15 min.

##### H&E

Post-fixed cells were immersed within hematoxylin (Sigma-Aldrich) solution for 15 min. After water washing, cells were immersed a few seconds within eosin (Sigma-Aldrich) solution. Then, cells were dehydrated through increasing alcohol solutions, clarified by xylene and finally mounted with a coverslip using DPX (Sigma-Aldrich) and observed using a Nikon Eclipse Ni light microscope (Nikon, Tokyo, Japan), equipped with a digital camera connected to the NIS Elements software for image analysis (Nikon, Tokyo, Japan).

H&E-positive cell counts were performed on one slide per experimental group under 20 × magnification, where 3 different microscopic fields were selected containing distinct, not overlapped cells. Cell counts were carried out by two independent investigators and values expressed as mean percentage ± SEM percentage (assuming controls as 100%). Data refer to three independent experiments.

##### FJ-B histofluorescence

Cells were fixed in a paraformaldehyde (4%) solution for 15 min, washed in PBS and incubated with 0.06% potassium permanganate (10 min, at 21 °C). After washing in distilled water, cells were incubated for 20 min in a FJ-B solution prepared by dissolving 0.01% FJ-B (Merck Millipore, Billerica, MA, USA) in acetic acid. Cells were incubated in 4 × 10^–4^% of FJ-B solution for 20 min and then cover-slipped with a mounting medium. FJ-B-positive cells were analyzed using a Nikon Eclipse Ni light microscope (Nikon, Tokyo, Japan), equipped with a florescence lamp and a digital camera connected to the NIS Elements software for image analysis (Nikon, Tokyo, Japan).

Cell were counted through the fluorescence microscope at 20 × magnification. The number of FJ-B-fluorescent cells was expressed as the mean number ± SEM for each experimental group. Data refer to three independent experiments counted by two independent investigators.

Adobe Photoshop CS4 Extended program (version 11.0, Adobe Systems Inc., San Jose, CA, USA) was used for artwork.

For immunohistochemistry, cells administered 3-MA (10 mM) or vehicle were permeabilized with 0.1% TritonX-100 (Sigma-Aldrich), This was followed by 1 h incubation in a blocking solution (to prevent non-specific binding of primary antibodies). Then, PC12 cells were incubated at 4 °C overnight within a solution containing primary antibodies anti-alpha-syn (Abcam, Cambridge, UK), anti-p62 (Life Technologies, Carlsabad, CA, USA), anti-Poly-ub (Abcam); anti-P20S (Abcam); anti-LC3-II (Abcam). All primary antibodies were diluted 1:100 in a PBS solution containing 1% normal goat serum. Then, cells were immersed for 1 h in the solution containing secondary fluorescent antibodies Alexa 488 (Life Technologies) or Alexa 594 (Life Technologies) diluted 1:200. These cells were transferred on coverslip and finally mounted with the medium Fluoroshield (Sigma-Aldrich). Cell nuclei were stained with the fluorescent dye DAPI (Sigma-Aldrich). Slides were observed under Nikon Eclipse Ni light microscope (Nikon) equipped with a fluorescent lamp and a digital camera connected to the NIS Elements Software for image analysis (Nikon). Detailed information concerning all primary and secondary antibodies used in these experiments, including catalogue number and RRID code, are reported in the Supplementary Information (Supplementary Table S1).

Merging areas of immunofluorescence were measured by using Image J software (NIH, Version 1.8.0_172, Bethesda, MD, USA) and they were expressed as mean percentage ± SEM where controls were considered as 100.

Densitometry of the LC3-II- and the p62-fluorescence was measured within the very same cell by using Image J software (NIH, Version 1.8.0_172, Bethesda, MD, USA). Data were expressed as the mean percentage ± SEM percentage of 60 cells per group, assuming 100% the intensity measured in controls.

Comparisons between groups were carried out by ANOVA with Sheffe’s *post-hoc* analysis; the null hypothesis H_0_ was rejected for *p* < 0.05.

### Transmission electron microscopy (TEM)

For TEM analysis PC12 were centrifuged at 1000×*g* for 5 min, they were rinsed in PBS, and fixed within paraformaldehyde 2.0%, and glutaraldehyde 0.1%, which were both diluted in a 0.1 M PBS solution, pH 7.4 for 90 min at 4 °C. After repeated washing in PBS (0.1 M), samples were further fixed in 1% osmium tetroxide (OsO_4_) for 1 h at 4 °C. Then, samples were dehydrated in gradient solutions of ethanol (30%, 50%, 70%, 90% and 95%, 5 min each, and 100%, 60 min). Finally, samples were embedded in epoxy-resin. The concentration of the fixing solutions, and the procedure to embed the samples within epoxy-resin, were validated in previous studies, where both plain and immuno-gold-based ultrastructural morphometry were carried out (Fornai et al. 2001a, b; Lazzeri et al. 2007). In fact, combining these aldehydes, OsO_4_, and epoxy-resin allows a minimal epitope covering (which is useful for stoichiometry counts with immuno-gold), while preserving sub-cellular architecture (which allows to define the specific organelles where counts were carried out in situ).

Ultra-thin (90 nm thick) slices were collected on nickel grids and they were used either for plain, or post-embedding immuno-gold electron microscopy.

### Plain electron microscopy

When plain electron microscopy was carried out, ultra-thin slices on nickel grids were stained with uranyl acetate (to enhance the contrast of nucleic acids and amino acids) and lead citrate (to enhance the staining of heavy metals). These samples used for plain electron microscopy were directly observed at Jeol JEM SX100 electron-microscope (Jeol, Tokyo, Japan).

### Immuno-electron microscopy

When stoichiometry antigen detection was needed, ultra-thin (90 nm thick) slices placed on nickel grids and they were layered on droplets of aqueous sodium metaperiodate (NaIO_4_), for 30 min, at 22 °C. This specific step was aimed at removing a potential excess of OsO_4_, which may cover antigen epitopes. In order to remove residual NaIO_4_, reiterated washing of the grid in PBS (three times, 5 min each) was carried out. At this point, grids were further layered on droplets containing a so-called blocking solution to prevent non-specific immunostaining (the blocking solution consists of 10% goat serum, which enables to access tissue through 0.2% saponin acting as a surfactant) components of the blocking solution were dissolved in PBS 1 M and persisted in contact with the grid for 20 min, at 22 °C. Other drops were prepared containing the primary antibody on which grids were further layered. This step occurred with specific conditions of humidity, at a temperature of 4 °C overnight. This drop-mediated incubation may involve single or double primary antibody(ies). In the present experiment the following drops were used: alpha-syn 1:100; p62, 1:100; Poly-ub 1:100, P20S 1:100. All primary antibodies reported below are the same used for the immunohistochemistry. The drops contained the antibody dissolved in ice-cold PBS solution also containing 1% goat serum and 0.2% saponin to improve antigen binding. The day after, grids were washed in cold PBS (three times, 5 min each). Thus, grids where ultrathin sections were layered, were further layered on drops containing gold-conjugated secondary antibodies. The size of gold particles varies specifically featuring either 10 nm or 20 nm diameter. All secondary, gold- conjugated antibodies were provided by BB International (Cardiff, UK). In the drop, the concentration of the secondary antibody was 1:100 within a blocking buffer (1% goat serum and 0.2% saponin) in PBS. Drops were exposed for 1 h, at 22 °C. At this point, after previous rinsing grids prepared for immuno-gold detection were layered on drops of 1% glutaraldehyde, for 3 min before being washed in distilled water to be exposed to the classic processing for plain electron microscopy, which consists of staining with uranyl acetate and lead citrate, as reported above. Ultra-thin sections were finally observed at TEM (JEOL JEM SX100). Control ultra-thin sections were incubated with secondary antibodies only. The number of immuno-gold particles relates stoichiometry to the number of molecules of each specific antigen (alpha-syn, p62, Poly-ub, P20S).

Detailed information concerning all primary and secondary antibodies used in these experiments, including catalogue number and RRID code, are reported in the Supplementary Information (Supplementary Table 1).

### Extended statistical analysis (procedures validation, sampling method, bias avoidance, description within each group and inference between groups)

The statistical relevance of combining various histochemical procedures (TB; H&E; FJ-B) to detect gross effects induced by various doses of the autophagy inhibitor 3-MA on cell death and cell pathology includes a number of issues. In fact, each method provides some specific information. For instance, the amount of severe cell damage assessed by H&E is based on actual lack of cell structures (uniquely visible among light microscopy procedures) and the presence of remarkable alterations. These concern abnormal cell size and shape, cytosol staining and nuclear staining frankly differing from standard control cells even during spontaneous degeneration. In this case the authentic counts rule out some cell degeneration, which also occur in control conditions. In keeping with H&E staining, the amount of cell alterations required to call a cell out of the viable range is severe and needs to be clearly evident at light microscopy, which may conversely reduce the count of irreversible cell damage when this is finely detected at sub-cellular level. The use of TB allows to establish the loss of membrane integrity as an index of cell damage. In fact, this method provides data based on the staining with a dye (TB) which penetrates the cell based on the loss of membrane permeability and/or integrity. In the case of FJ-B, we still lack an in-depth knowledge of which and how many markers are responsible for FJ-B-induced fluorescence. In this context, non-pathological staining may be induced by some molecules, which target FJ-B providing some false positive results. This potential bias may lead to non-degeneration dependent FJ-B positivity. This may explain why cell damage detected by FJ-B-staining is the highest compared with all other methods. The relevance of autophagy for the expression of markers stained by FJ-B is presently unknown, which also leaves other question marks on data interpretation. Nonetheless, the dose–response curve for 3-MA provides an evidence, which is quite similar for all histochemical measurements, this contributes to the validation of cell damage independently by the kind of method being considered. The integration of three different approaches and the general consistency, concerning the detrimental effects of various doses of 3-MA, is statistically relevant to infer on the dose–response curve for 3-MA-induced degeneration. In particular, FJ-B was never used so far to mark degeneration following autophagy inhibition, the present data inherently validates FJ-B as a marker of autophagy-dependent neurodegeneration. The histochemical data were similarly treated in descriptive statistics. In detail, for TB histochemistry viable and non-viable cells were counted under reversed microscopy, and cell death was expressed as the mean percentage ± SEM of TB-positive cells out of total cells in the same chamber. Data from each group are reported as the mean of three separate counts in different though equivalent chambers. These three independent experiments were counted by two blind investigators. For H&E, viable H&E-stained cells in each experimental group were counted and they were expressed as mean percentage ± SEM of control (corresponding to 100%). Data refer to three independent experiments. When considering viable H&E-stained cells an important issue needs to be clarified since dramatic alterations of cell morphology such as faint cells, highly vacuolated cells, and disrupted cell membranes rule out the inclusions in the count of viable cells.

When comparing the effects of various doses of 3-MA and controls the inference was based on ANOVA with Sheffè’s *post-hoc* analysis considering unlikely the null hypothesis H_0_ when *p* < 0.05.

For the semi-quantitative assessment of the autophagy flux, the intensity of the LC3-II- and the p62-fluorescent signal was measured within the very same cell by using Image J software (NIH, Version 1.8.0_172, Bethesda, MD, USA) and was reported as the mean percentage ± SEM percentage of 60 cells per group, assuming 100% the intensity measured in controls. Comparison between groups were carried out by ANOVA with Sheffe’s *post-hoc* analysis; the null hypothesis H_0_ was rejected for *p* < 0.05. For immunohistochemistry, descriptive analysis for each immunostaining was based on the surface of the immunopositive area in each cell group. This area was reported as a percentage of the area measured in control (100%) to provide a sudden comparison for all antigens. Areas of immunofluorescence were defined by using Image J software (NIH, Version 1.8.0_172, Bethesda, MD, USA). Comparisons between groups were carried out by ANOVA with Sheffe’s *post-hoc* analysis; the null hypothesis H_0_ was rejected for *p* < 0.05.

The real size of the area was also counted and it was expressed in µm^2^ as reported in Table 1. These data were reported as the mean ± SEM of 90 cells per group (30 cells for each experiment carried out in triplicate). The inference concerning different percentage and different absolute numerical values between groups was carried out by using one way ANOVA with Sheffe’s *post-hoc* analysis. Adobe Photoshop CS4 Extended program (version 11.0, Adobe Systems Inc., San Jose, CA, USA) was used to create the artwork.

**Table 1:** Immunofluorescence areas for each antigen observed at light microscopy were measured in µm^2^ in control cells and cells administered 3-MA (10 mM)

	CONTROL	3-MA
alpha-syn	14.1 ± 0.5	21.4 ± 0.6*
p62	7.3 ± 0.3	42.4 ± 1.2*
poly-ub	9.8 ± 0.5	57.2 ± 1.8*
P20S	24.2 ± 1.2	29.3 ± 0.9

Values are expressed as mean area ± SEM

*p<0.05 compared with controls

At any rate, the semi-quantitative non-linear distribution of immunostained areas and antigen amounts remain a limit inherent to immunohistochemistry, which moved the present study towards the achievement of a stronger statistical power provided by sub-cellular stoichiometry. Thus, during sub-cellular analysis at TEM, areas were sampled based on the accumulation of specific antigens such as alpha-syn, p62 and Poly-ub within each antigen-rich domain averaging 2 µm^2^ cell area. Thus, these areas, where clusters of immunogold were mostly represented and were selected for in situ stoichiometry. In this way, the amount of each antigen (alpha-syn, p62, Poly-ub, P20S) represents the authentic number of protein molecules deriving from a 1:1 ratio between a protein and an immunogold particle, which allows in situ quantification according to specific antigen stoichiometry. This approach allows a quantitative measurement of specific protein amount within the cell densest spots (2 µm^2^) providing reliable values counted in situ (Bergensen et al. 2008).

Counts of immunogold particles (either 10 nm and/or 20 nm) were carried out at TEM by using a magnification (8000 ×), which allows to recognize concomitantly immuno-gold particles along with cell organelles for in situ stoichiometry under the same magnification. Descriptive values for each antigen were expressed as the mean ±  SEM within selected areas from n = 30 cells per group (3MA-treated cells and controls). The 2 µm^2^ area was also quantified concerning non-protein tubulo-vesicular structure, which is intended as the areas covered by membranous organelles and vesicular structures (tubulo-vesicular membranes). Comparisons were carried out by ANOVA with Sheffe’s post-hoc analysis.

Comparison between alpha-syn, p62, Poly-ub and P20S immuno-gold were carried out by using ANOVA with Sheffe’s post-hoc analysis; the null hypothesis H_0_ was rejected for p < 0.05.

## Results

### Dose–response effects of 3-MA on cell viability

**Fig. 1:**
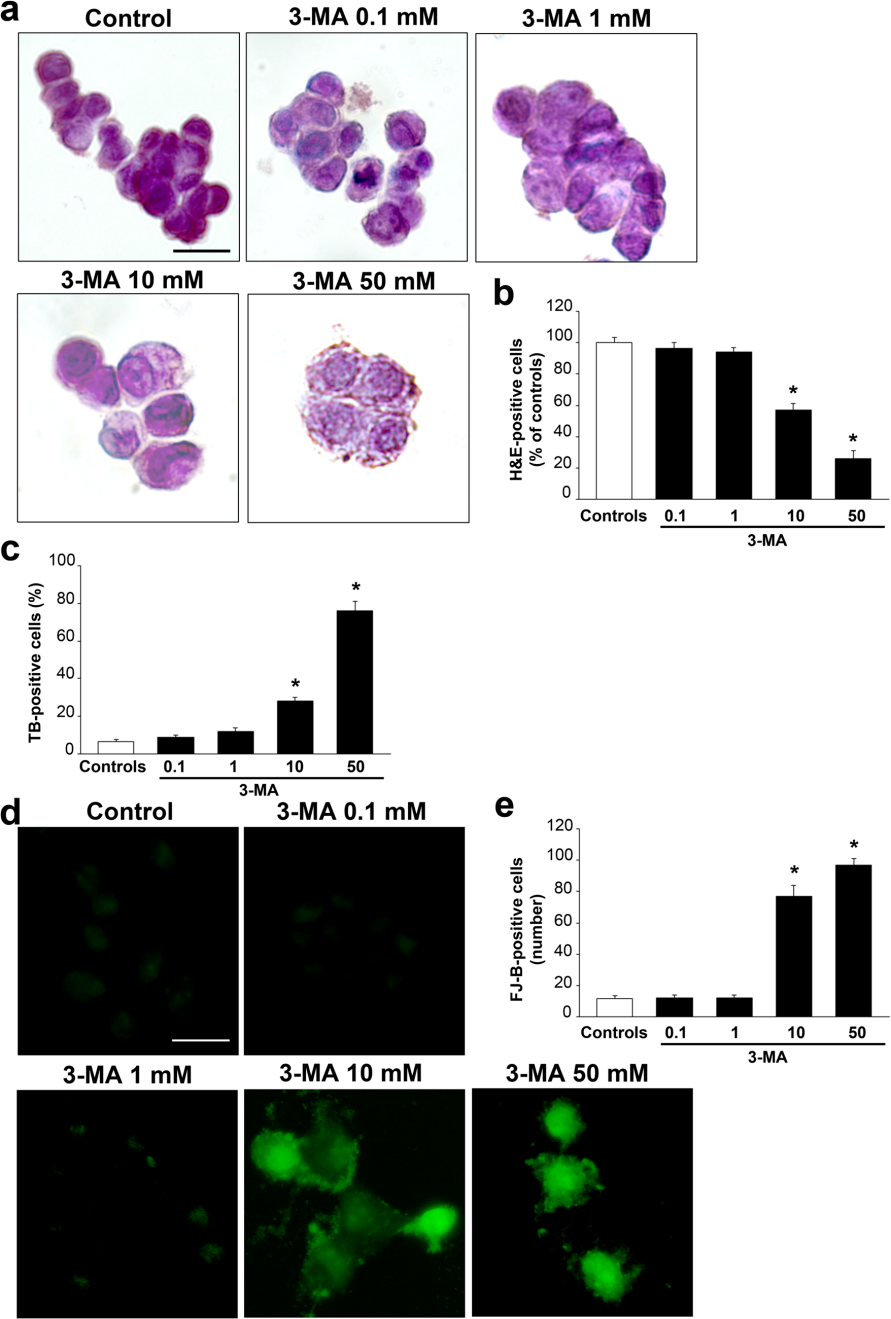
3-MA dose-dependently increases cell damage. **a** Representative pictures of H&E-stained PC12 cells from controls and following increasing doses of 3-MA (0.1 mM, 1 mM, 10 mM, 50 mM). The graph in **b** reports the counts of viable H&E-positive cells, expressed as percentage of controls. **c** The graph reports the percentage of TB-positive cells from controls and increasing doses of 3-MA (0.1 mM, 1 mM, 10 mM, 50 mM). **d** Representative pictures of FJ-B-stained cells from controls and increasing doses of 3-MA (0.1 mM, 1 mM, 10 mM, 50 mM). The total number of FJ-B-positive cells is reported in graph (**e**). *p < 0.05 compared with controls. Scale bars: 14 µm (**a**, **d**)

As shown in representative pictures of Fig. 1a, the autophagy inhibitor 3-MA produces dose-dependently cell loss and cell damage of catecholamine cells, when documented by H&E staining. In detail, the doses of 0.1 mM and 1 mM 3-MA do not alter noticeably the percentage of cell number and cell shape, cell size and cell morphology. In contrast, higher 3-MA doses, starting from 10 mM produce an evident decrease in cell density. In these conditions, spared cells undergo dose-dependent alterations concerning their size, which was enlarged, their shape, which was roundish, the occurrence of cytosolic vacuoles and a decrease in cysotolic eosinophilia, which suggests a loss of cell architecture. In detail, the highest dose of 50 mM 3-MA produces faint cells with non-structured content including both nucleus and cytosol (Fig. 1a). These representative pictures are consistent with the occurrence of non-viable cells, which is reported in the graph of Fig. 1b, where a significant loss of viable H&E stained cells starts to be evident for the dose of 10 mM 3-MA, and approaches a loss of roughly 75% for the highest dose of 50 mM 3-MA. Similarly, the penetration of TB within the cell, which occurs when cells are damaged, is clearly evident from the dose of 10 mM 3-MA as reported in the graph of Fig. 1c. The highest dose of 50 mM 3-MA leads to almost 80% of TB-stained cells, which matches the amount of cell damage assessed following H&E (compare Fig. 1b and c).

When the histofluorescent dye FJ-B was applied, the number of histofluorescent, presumably degenerating cells, increased progressively with increasing 3-MA doses as evident in representative pictures of Fig. 1d. In detail, the 3-MA 0.1 and 1 mM doses do not produce any noticeable fluorescence. Instead, fluorescence is clearly evident for the dose of 10 mM 3-MA and 50 mM 3-MA. As reported in the graph of Fig. 1e, the doses of 0.1 and 1 mM sorted a fluorescence, which was rarely detectable as occurring in controls. In contrast, the dose of 10 mM produces an average of 70 fluorescent cells per slide, while 50 mM 3-MA produces fluorescent cells over of 95 per slide. Although not differing markedly compared with H&E and TB, this highest value suggests that, as reported in extended statistics, even autophagy-dependent molecules may contribute to FJ-B activation or, alternatively FJ-B is very sensitive as a marker of degeneration. At any rate, these slight discrepancies do not alter the significance of the data. The crucial point of this series of experiments was to select the best dose of the autophagy inhibitor 3-MA in order to produce significant degeneration still in the presence of spared cells, where cytopathology could be detected. In this context, the dose of 3-MA 10 mM appears the most suitable to proceed with immunohistochemistry and immunogold and subcellular analysis.

### Immunohistochemistry

In preliminary experiments, the autophagy flux was measured to assess the effectiveness of 3-MA in inhibiting autophagy at the dose (10 mM) and time interval (72 h) selected in this experimental setting. Representative LC3-II- and p62-immunofluorescent pictures of Supplementary Figure S1a show that the immunofluorescence for LC3-II and p62 is increased in cells treated with 3-MA compared with vehicle-treated cells (controls). Such an increased fluorescence is similar to that observed in cells treated with the autophagy inhibitor bafilomycin A1 for both the antigens (Supplementary Figure S1a). The graphs reporting the densitometry of the LC3-II and p62 immunofluorescence measured within the very same cell confirm that treatment with 3-MA produces the very same increases in LC3-II and p62 immunofluorescence than those produced by the autophagy inhibitor bafilomycin A1 (Supplementary Figs. 1b and 1c). These results demonstrate that 3-MA, at the dose of 10 mM and allowed to act for 72 h, is an effective autophagy inhibitor.

**Fig. 2:**
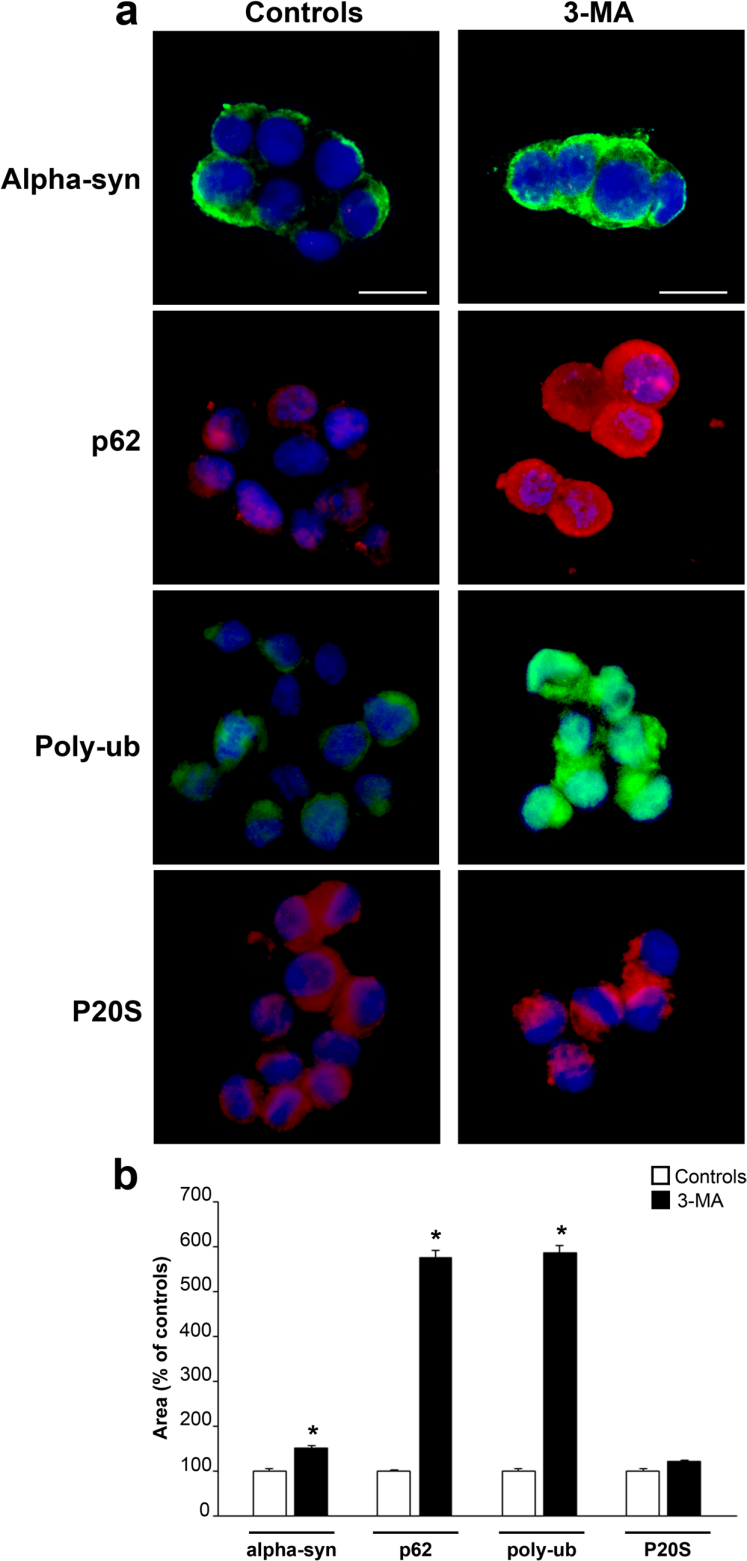
The increase of p62 and Poly-ub exceeds the increase of alpha-syn following 3-MA. **a** Representative immunofluorescence of each protein from control cells (administered medium); and following 3-MA (10 mM) dissolved in the medium. These pictures show an increase in all four antigens following 3-MA. In detail, such an increase is dramatic for p62 and Poly-ub, while alpha-syn undergoes a moderate increase and the effects on P20S immunofluorescence are negligible. These representative images are quantified in graph (**b**) reporting the mean area of immunofluorescence per cell for each antigen. This is expressed for 3-MA-treated cells as a percentage of controls. Remarkably, 3-MA produces an increase of either p62 or Poly-ub, (roughly six-folds of controls). The increase in alpha-syn is significant, though it is much less pronounced (roughly 150% of controls), while P20S does not change noticeably. The numerical expression of each immunofluorescent area is reported in Table 1. *p < 0.05 compared with controls. Scale bars: 17 µm

In Fig. 2a representative pictures report immunofluorescence for the gold-standard marker of LBs alpha-syn along with immunostaining for some proteins involved in protein clearing pathways such as p62 (sequestosome), Poly-ub, and P20S following exposure of cell cultures to the autophagy inhibitor 3-MA at the dose selected from the dose–response study (10 mM 3-MA). As evident from these pictures in control conditions immunofluorescence is slightly more evident for alpha-syn and P20S. This is in sharp contrast with pictures reporting these antigens following 3-MA administration. In fact, as shown in the right row of Fig. 2a, a striking difference is produced concerning the expression of p62 and Poly-ub, which far exceeds the increase in alpha-syn and P20S. Specifically, alpha-syn slightly increases, while P20S does not appear to be influenced by 3-MA administration. In Table 1 and graph of Fig. 2b these effects are reported by counting the immunofluorescent areas for each antigen both in control and 3-MA conditions. In detail, the authentic areas are reported in Table 1 and they are also presented as a percentage of control to emphasize the effects produced by 3-MA (graph of Fig. 2b). In detail, as reported in the graph of Fig. 2b, the increase of immunofluorescent area for both p62 and Poly-ub is similar (roughly 600% of controls) while the increase in alpha-syn produced by 3-MA exposure is roughly four-folds less pronounced (150% of controls). In the case of P20S, administration of 3-MA does not produce any noticeable change (Fig. 2b).

### Immunogold stoichiometry

**Fig. 3:**
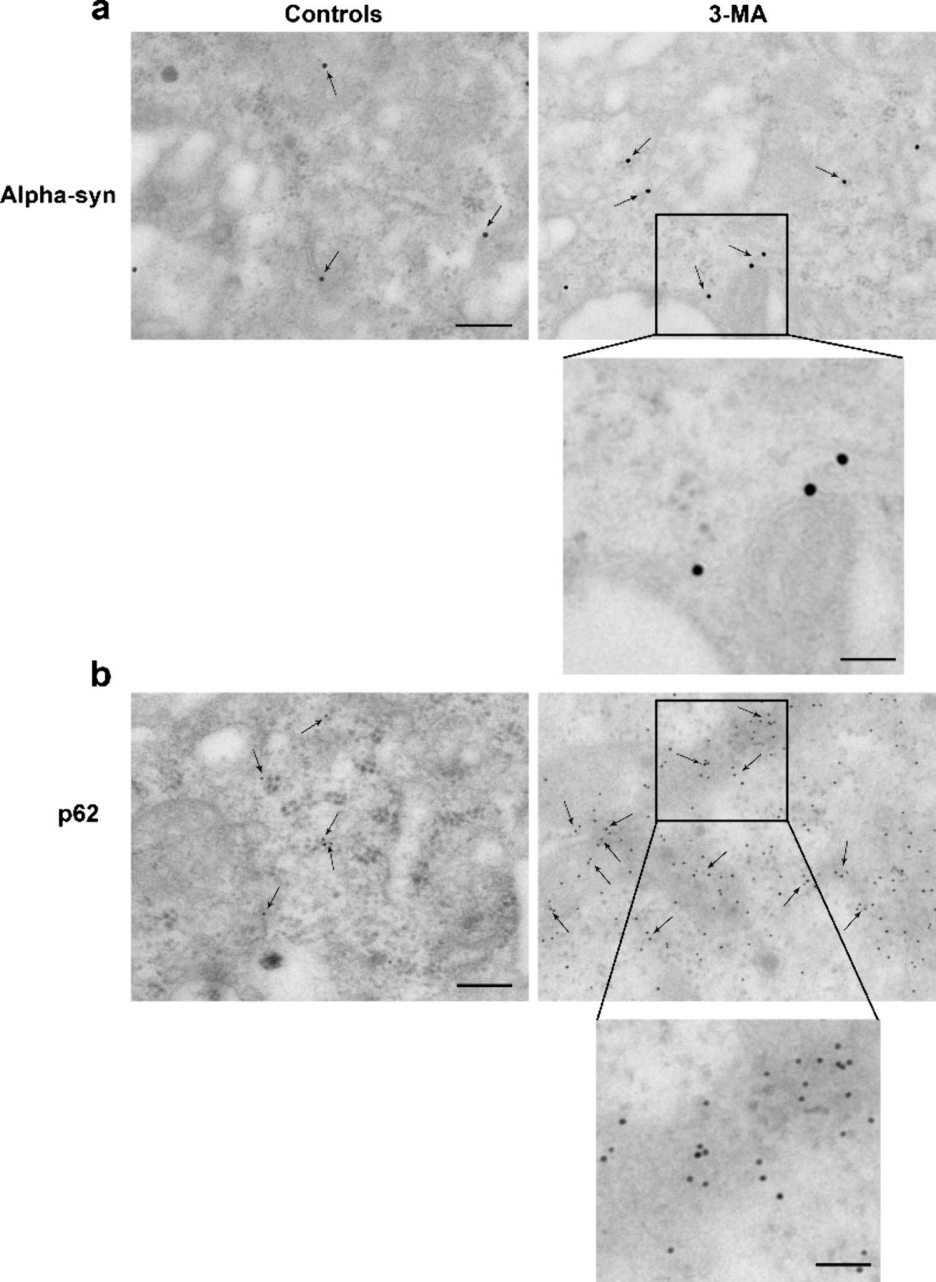
Dramatic increase of p62 compared with alpha-syn at immunoelectron microscopy. When the effects of the autophagy inhibitor 3-MA are observed at TEM following staining with immunogold, the increase of alpha-syn (representative pictures in **a**) is way less pronounced compared with the occurrence of p62 immunogold-stained molecules (representative pictures in **b**). This is further evidenced by increasing magnification as reported in the inserts of both** a** and **b**. Arrows indicate each specific immunogold-stained antigen. Scale bars: 255 nm (**a**, **b**); scale bars in both inserts: 114 nm

**Fig. 4:**
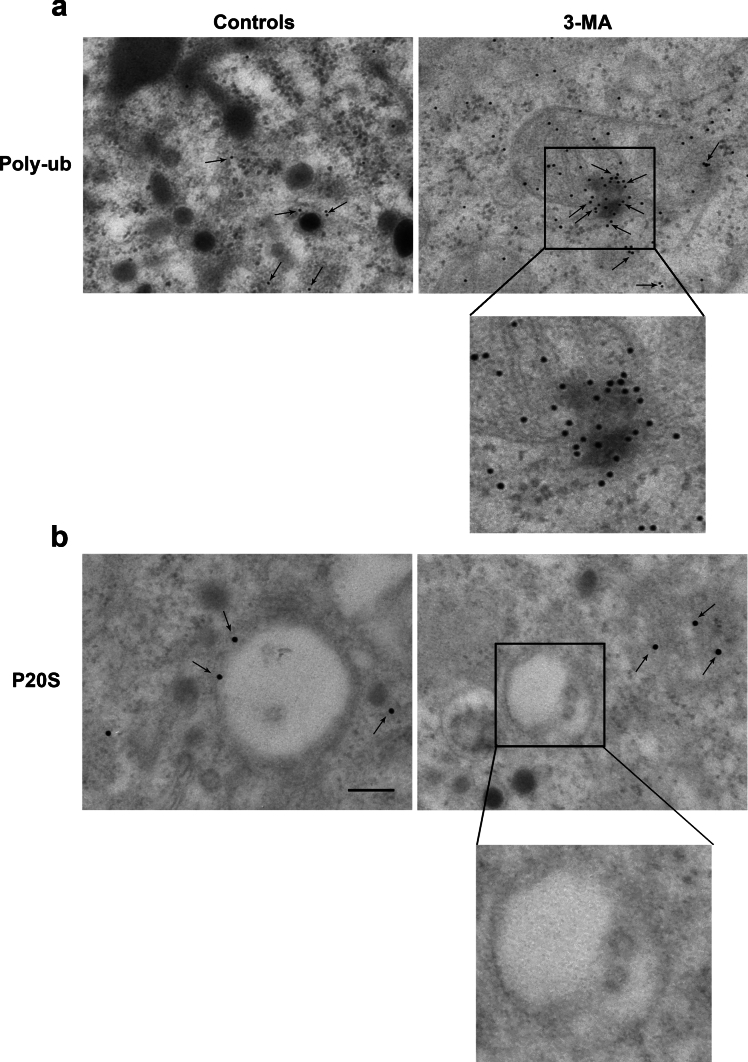
Dramatic increase of Poly-ub compared with P20S at immunoelectron microscopy. When the effects of the autophagy inhibitor 3-MA are observed at TEM following staining with immunogold, the increase of Poly-ub (representative pictures in **a**) is way more pronounced compared with the occurrence of P20S immunogold-stained molecules (representative pictures in **b**). This is further evidenced by increasing magnification as reported in the inserts of both** a** and **b**. Arrows indicate each specific immunogold-stained antigen. Scale bars: 255 nm (**a**, **b**); scale bars in both inserts: 114 nm

In order to detect in situ the structure of specific cytosolic protein clusters following pharmacological autophagy inhibition specific domains were selected. As reported in the extended statistics, the sampling was based on sites and areas which possess the densest amount of each single protein under investigation. The sampling of various cytosolic areas led to select cell domains measuring an area of 2 µm^2^. Within these spots, specific immunoelectron microscopy was carried out by using either 10 nm or 20 nm immunogold to measure the density of each antigen. This was referred to both as total amount in control and 3-MA, and percentage of 3-MA compared with controls. As reported in representative pictures of Figs. 3 and 4 immunogold was visible in situ within specific cytosolic compartments allowing to establish the amount and preferential cytosolic placement.

In detail, pictures of representative Fig. 3a show immunogold for alpha-syn in a control cell and following 3-MA at various magnifications. A slight increase is evident following 3-MA compared with control conditions. In Fig. 3b, in the same experimental conditions the occurrence of p62 is representatively reported. The occurrence of p62 appears slightly more evident in controls and it is massively increased following 3-MA. A similar result was obtained for the antigen Poly-ub, which is reported in representative pictures of Fig. 4a, where it is evident the dramatic effect produced by autophagy inhibition, which determines the clustering of innumerous immunogold particles. In sharp contrast, variations in the amount of P20S were not evident (representative Fig. 4b).

**Fig. 5:**
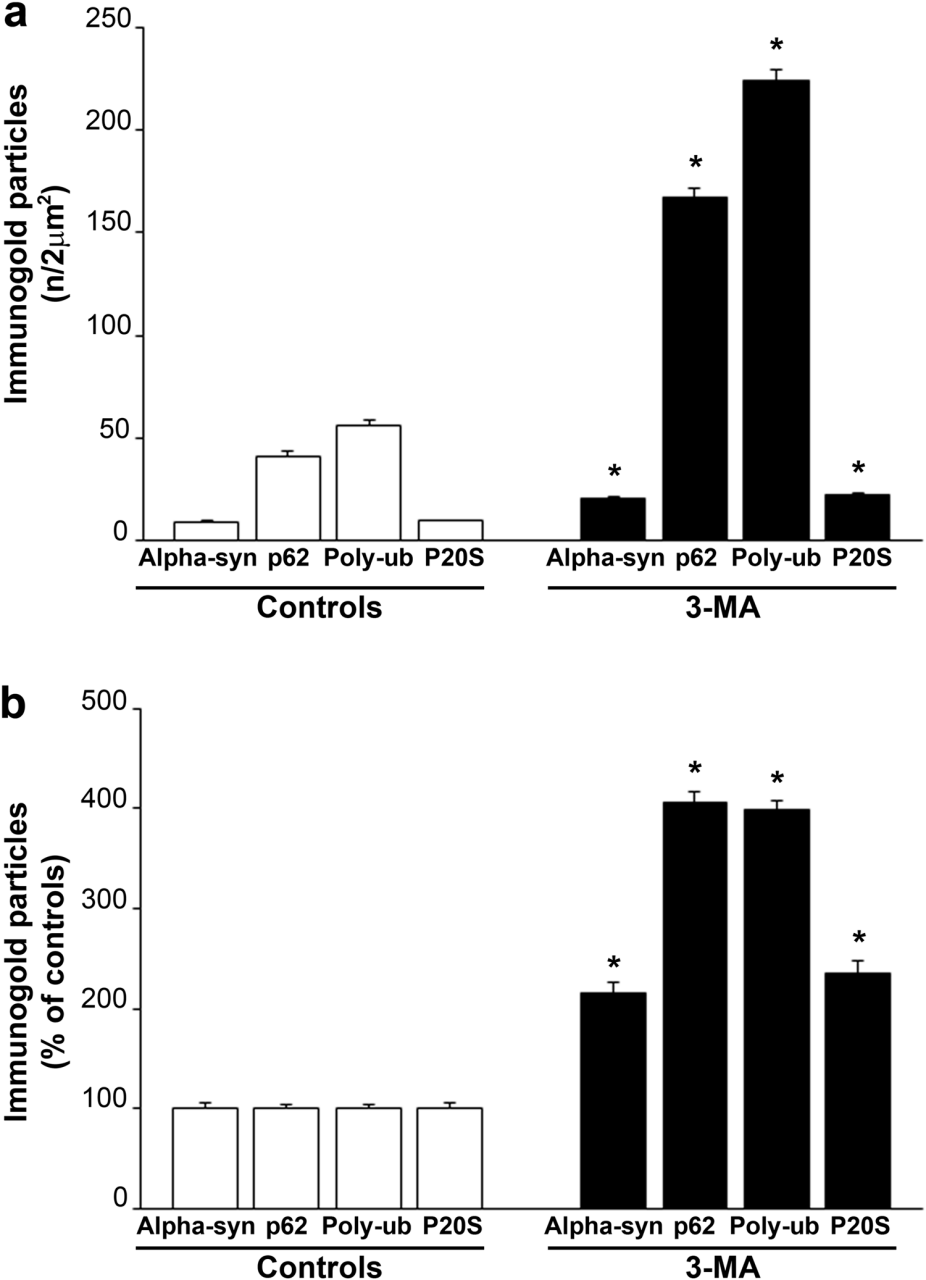
Counts of increase in p62 and Poly-ub largely exceed the counts of alpha-syn and P20S. **a** Authentic counts of stoichiometry amount of absolute particles of alpha-syn, p62, Poly-ub, and P20S following the autophagy inhibitor 3-MA 10 mM, compared with controls. **b** Percentage of the amount of alpha-syn, p62, Poly-ub, and P20S following the autophagy inhibitor 3-MA 10 mM, considering controls for each antigen as 100%. Authentic counts are expressed as the number of immunogold particles counted in each highly clustered domain measuring a surface of 2 µm^2^. The size of this domain was sampled based on a random distribution count (reported in extended statistics). Each count was carried out in 50 cells. *p < 0.05 compared with each specific control

The graphs of Fig. 5 report the counts of each antigen in its densest spot. For each antigen, the amount of immunogold particles was expressed both as authentic amounts of molecules (Fig. 5a) and as a percentage of controls (Fig. 5b). In detail, the increase in alpha-syn following 3-MA was significant with 20 ± 0.9 immunogold particles/2 µm^2^ compared with 9.2 ± 0.5 immunogold particles/2 µm^2^ of controls. Such a duplication in the amount of alpha-syn produced by autophagy inhibition is consistent with the metabolism of alpha-syn through the autophagy pathway. Remarkably, when the counts of immunogold were measured for p62, the increase following 3-MA was impressive, with 167.5 ± 4.2 immunogold particles/2 µm^2^ compared with 41.3 ± 1.8 immunogold particles/2 µm^2^ of controls, which corresponds to a four-folds increase of p62. This was parallel to the increase counted for Poly-ub which rises from 56.2 ± 2.5 immunogold particles/2 µm^2^ of controls up to 224.4 ± 5.0 immunogold particles/2 µm^2^ following 3-MA. Remarkably, we noticed that the cytosolic domains where p62 and Poly-ub were mostly increased were similar and possessed a similar ultrastructure featuring abundant tubulo-vesicular elements. This is quite different from the spots where alpha-syn is more abundant. Although the amount of P20S was steady in controls and following 3-MA, we noticed a trend for P20S to dissipate from tubulo-vesicular membranes under the effects of 3-MA.

**Fig. 6:**
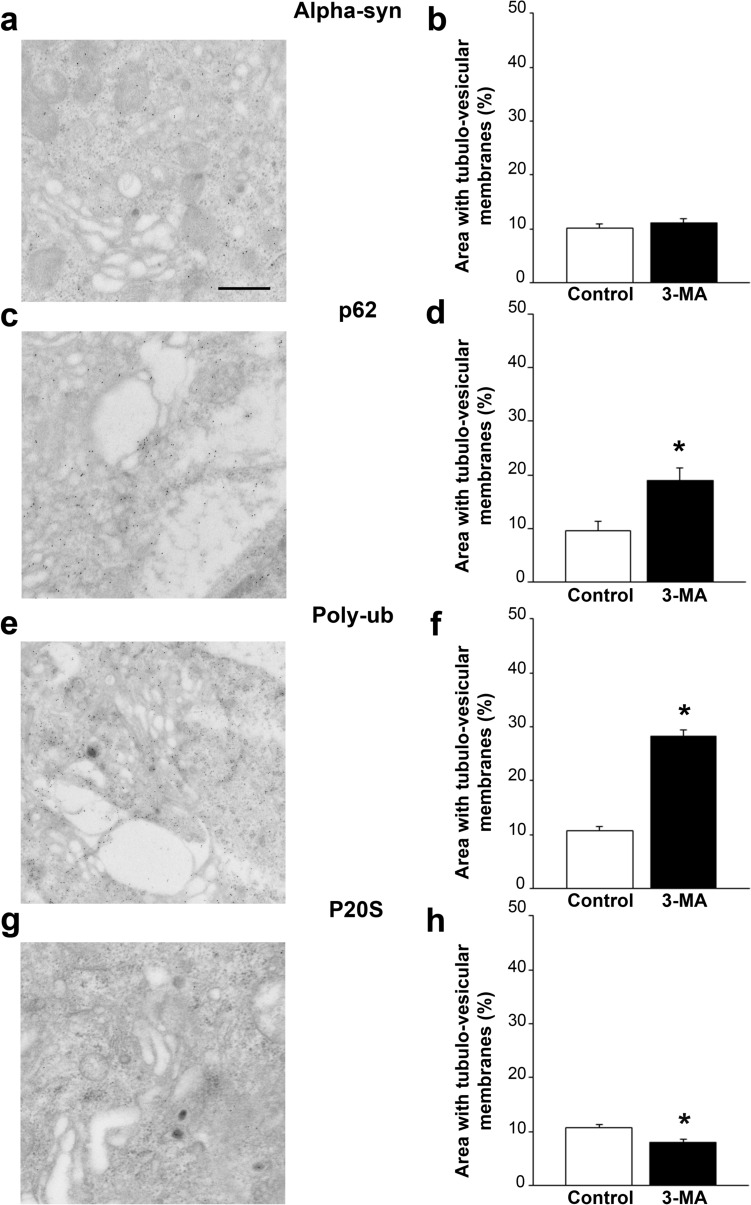
Poly-ub- and p62-rich areas are composed of abundant tubulo-vesicular structures. Representative pictures and graphs showing and reporting the amount of tubulo-vesicular areas in the proximity of clustering of specific antigens. Alpha syn (**a**; **b**); p62 (**c**; **d**); Poly-ub (**e**; **f**); P20S (**g**; **h**) are reported considering the picture and the graph, respectively. The association between antigen clustering and occurrence of tubulo-vesicular structures reaches the maximum following staining for both p62 and Poly-ub (**c–f**). In contrast, no structural variations were measured compared with surrounding cytosol concerning those areas where alpha-syn clustering was considered (**a**; **b**). A paradox decrease of these tubulo-vesicular structures was counted in association with P20 antigen (**g**; **h**). Areas considered for the counts correspond exactly to that reported in the representative image covering a surface of 2 µm. Each count was carried out in 50 cells. *p < 0.05 compared with each specific control. Scale bars: 220 nm

In Fig. 6a a representative picture reports the occurrence of alpha-syn densest spots aside from the area of tubulo-vesicular structures which is reported in the graph of Fig. 6b reporting an amount of tubulo-vesicular area following 3-MA, which is similar to controls within the 2 µm^2^ alpha-syn rich spots. In Fig. 6c a representative picture reports the occurrence of p62 densest spots, which largely correspond to an area featuring tubulo-vesicular structures. This is reported in the graph of Fig. 6d reporting tubulo-vesicular areas following 3-MA which are way in excess compared with controls within the 2 µm^2^ p62 rich spots. In Fig. 6e a representative picture reports the occurrence of Poly-ub-densest spots, which overlap tissue structures with p62-rich spots, meaning that Poly-ub-rich areas largely overlap with tubulo-vesicular structures. This is reported in the graph of Fig. 6f.

In Fig. 6g a representative picture reports the occurrence of P20S densest spots, consisting of a few immunogold particles. Interestingly, while in controls these may be found in the lumen of tubulo-vesicular structures, following 3-MA such a placement was never detected (graph of Fig. 6h).

## Discussion

Lewy bodies are routinely considered alpha-syn-based neuronal inclusions. In line with this, in PD the role of alpha-syn is considered to be essential for disease onset and progression. However, evidence indicates that LBs are composed of a number of proteins other than alpha-syn. Moreover, non-protein structures are increasingly recognized as a significant component of LBs, both ex vivo from PD and DLB patients or in vitro from cell cultures. Therefore, it is not surprising that some findings lead to reconsider the assumption that alpha-syn is essential for LB structure and seeding as well as for PD cytopathology (Forno 1996; Iwasubo et al. 1996; Shahmoradian et al. 2019; Lashuel 2020; Estaun-Panzano et al. 2023; Lenzi et al. 2024). The present research work stemmed from the evidence that natural cytopathology of PD is rather the consequence of a dysfunctional cell clearance within catecholamine neurons. In turn, this may produce the accumulation of a number of proteins and organelles, which are no longer cleared from the cell. Such a dysfunction in cell clearance mostly concerns altered autophagy. In fact, PD patients feature altered autophagy within spared cells of the *substantia nigra* (Anglade et al. 1997), while specific mutations of genes involved in the autophagy pathway produce experimental PD and familial PD in humans (Ferrucci et al. 2008; Pasquali et al. 2009; Lu et al. 2020; Almeida et al. 2020; Singh and Muqit 2020; Gordevicius et al. 2021; Teixeira et al. 2021; Dehestani et al. 2022; Zhang et al. 2022; Kinet and Dehay 2023; Hattori et al. 2024; Lenzi et al. 2024). Lending substance to this working hypothesis, when autophagy genes such as ATG7 are knocked out experimental PD occurs, where LBs are evident (Komatzu et al. 2006, 2007). The role of autophagy inhibition appears to be site-specific since, when ATG7 is selectively knocked out within mesencephalic DA neurons, LBs and PD are present as shown by Ahmed et al. (2012). Thus, to validate the role of autophagy in PD cytopathology, the present study analyzed whether a pharmacological inhibition of autophagy produced by 3-MA could lead to similar effects (i.e. catecholamine cell death and PD-like cytopathology). As expected, here we provide evidence that the autophagy inhibitor 3-MA dose-dependently produces massive cell loss in vitro within catecholamine cell cultures. This effect is evident at light microscopy for the doses of 10 mM and 50 mM 3-MA as assessed by the concomitant use of various histochemical procedures (H&E, TB, and FJ-B). It is remarkable that, apart from slight differences, all these procedures lead to similar dose–response curves. These findings confirm the seminal role of the autophagy pathway in promoting the survival of catecholamine neurons and provide the evidence that FJ-B may be used reliably to assess cell degeneration following autophagy inhibition. Based on these dose–response studies the dose of 10 mM 3-MA was selected to analyze variations of immunostaining for specific proteins, which are relevant for cell clearing pathways and represent constituents of LBs such as alpha-syn, p62, Poly-ub, and P20S. In fact, 10 mM 3-MA does not produce massive cell death and a conspicuous number (more than a half) of catecholamine cells are spared. This allows both to analyze variations of immunostaining and, most importantly, to assess alterations of subcellular morphology and antigen counts through in situ stoichiometry according to Bergensen et al. (2008). Although 3-MA is commonly used as autophagy inhibitor, it should be considered that 3-MA may inhibit both class I phosphoinositide 3-kinase enzymes (PI3Ks) and class III phosphatidylinositol 3-kinase enzymes (PtdIns3Ks) (Petiot et al. 2000). This dual activity of 3-MA may produce opposite effects on autophagy. In fact, by acting on class I enzymes, 3-MA activates autophagy, while acting as inhibitor of class III enzymes 3-MA inhibits autophagy (Petiot et al. 2000; Wu et al. 2010; Klionsky et al. 2021). In most cases 3-MA effectively blocks autophagy, since class III enzymes act downstream to the class I enzymes. However, under specific experimental conditions, a few studies report that the activity of 3-MA on class I and class III enzymes may have different temporal patterns of inhibition, which may sort transiently an activation of autophagy (Wu et al 2010; Klionsky et al. 2021). To rule out such a pitfall, in the present study we actually assessed the autophagy flux by measuring the immunofluorescence for LC3-II and p62. We found that 3-MA, at the dose of 10 mM and for 72 h, inhibits autophagy in the present experimental conditions. In fact, a similar increase in LC3-II and p62 immunofluorescence was produced by 3-MA and the autophagy inhibitor bafilomycin A1. These data are in line with several previous papers carried out in catecholamine cells and confirm the common use of 3-MA as autophagy inhibitor (Castino et al. 2008, 2010; Chen et al. 2024). Immunohistochemical data reported here suggest that, at short time intervals following autophagy inhibition accumulation of alpha-syn is much less pronounced compared with p62 and Poly-ub. This confirms recent findings (Komatzu et al. 2007; Geisler et al. 2010; Sato et al. 2018, 2021). No significant change is detectable for the proteasome antigen P20S. Early accumulation of p62 and Poly-ub compared with alpha-syn confirms data obtained by several recent investigations both in experimental PD and some PD patients. In fact, p62- and Poly-ub-positive eosinophilic inclusions reminiscent of LBs may develop in experimental PD in alpha-syn knocked-out mice (Paine et al. 2013). Even when the alpha-syn gene is normally expressed, occurrence of p62 and Poly-ub within eosinophilic inclusions may anticipate the presence of alpha-syn (Komatzu et al. 2007; Geisler et al. 2010; Sato et al. 2018, 2021). In line with this, some evidence emphasizes the primary role of p62 and Poly-ub (Forno 1996; Iwasubo et al. 1996; Komatzu et al. 2007; Geisler et al. 2010; Sato et al. 2018, 2021). Recently, these proteins were suggested to seed LBs in experimental PD and some genetic forms of PD in humans (Komatzu et al. 2007; Hattori and Mizuno 2017; Sato et al. 2018, 2020; Gao et al. 2022; Noda et al. 2022; Oh et al. 2022; Ferrucci et al. 2024; Lenzi et al. 2024). In the present study, the rough assessment of protein amounts at immunohistochemistry was validated by stoichiometry counts carried out in situ. The data obtained by TEM further confirm and strengthen the evidence obtained in immunohistochemistry. In fact, following administration of the autophagy inhibitor 3-MA, a prominent accumulation of clusters of p62 and Poly-ub is documented here by immunoelectron microscopy. In detail, accumulation of both p62 and Poly-ub occurs way in excess compared with a moderate increase of alpha-syn, which can be measured following autophagy inhibition. Stoichiometry counts of immunogold particles demonstrate that electron microscopy discloses much more effectively than light immunocytochemistry the increase in p62 and Poly-ub compared with alpha-syn in the course of autophagy inhibition. The presence of dense clusters of both p62 and Poly-ub immunogold-stained particles typically occurs within similar areas of the cells, which differ from the sites where a moderate increase of alpha-syn was documented. These p62- and Poly-ub-rich cell domains were characterized by wide areas of tubulo-vesicular structures compared with surrounding cytosol. Conversely, the sites where alpha-syn moderately increases do not contain such an area of tubulo-vesicular structures, which differs significantly from surrounding cytosol. These findings are reminiscent of quite recent evidence provided by Shahmoradian et al. (2019) that, LB structure is richly composed of membranous vesicles and tubules, where autophago-lysosomal organelles are abundant. Indeed, such a description was pioneering carried out by Iwatsubo et al. (1996), who characterized ex vivo from PD patients the ultrastructure of LBs isolated through centrifuged fractions in a sucrose gradient. The pellet presumably corresponding to LBs typically stained for Poly-ub in the middle of membranous vesicles. These pioneer findings did not report the comparative stoichiometry counts of Poly-ub-rich vesicular areas as carried out in the present study. As a matter of fact, at that time the relevance of alpha-syn was not claimed yet, which explains the specific approach of Iwatsubo et al. (1996) to emphasize the occurrence of Poly-ub, rather than ubiquitin within LBs. The present study leads to the same data by measuring the rich density of Poly-ub, within specific cytosolic areas rich in tubulo-vesicular structures. These findings are also in line with recent evidence by Shahmoradian et al. (2019) showing that LB pathology in PD consists of crowded organelles and lipid membranes presumably deriving from the autophago-lysosomal compartment. Contemporary evidence emphasized how dysfunctional autophagy may be the key point to comprehend the pathophysiology of PD and LB composition. The conclusions of the present study confirm that pharmacological inhibition of the autophagy pathway leads to catecholamine cell degeneration, with subcellular alterations reproducing the structure and the nature of LBs. The chance to describe this phenomenon in vitro allowed us to infer the authentic relevance of proteins other than alpha-syn such as p62 and Poly-ub, which could be measured through in situ stoichiometry. Within these clusters of p62 and Poly-ub, tubulo-vesicular components occur in excess. This evidence confirms the relevance of an autophagy impairment both in the pathophysiology of PD and the seeding of LBs, while the prominent role of alpha-syn should be probably toned down at least at early time intervals.

## Data Availability

The data that support the findings of this study are available from the corresponding author upon reasonable request.
